# CtIP fusion to Cas9 enhances transgene integration by homology-dependent repair

**DOI:** 10.1038/s41467-018-03475-7

**Published:** 2018-03-19

**Authors:** M. Charpentier, A. H. Y. Khedher, S. Menoret, A. Brion, K. Lamribet, E. Dardillac, C. Boix, L. Perrouault, L. Tesson, S. Geny, A. De Cian, J. M. Itier, I. Anegon, B. Lopez, C. Giovannangeli, J. P. Concordet

**Affiliations:** 1 Museum National d’Histoire Naturelle, INSERM U1154, CNRS UMR 7196, Sorbonne Universités, 43 rue Cuvier, Paris, F-75231 France; 2 Translational Sciences, Sanofi, 13 Quai Jules Guesde, F-94400 Vitry-sur-Seine, France; 3 Centre de Recherche en Transplantation et Immunologie UMR1064, INSERM, Université de Nantes, CHU de Nantes, 30 Avenue Jean Monnet, F-44093 Nantes, France; 4 Equipe Labellisée Ligue Contre le Cancer, Institut de Cancérologie Gustave-Roussy, Université Paris-Saclay, CNRS UMR 8200, 114 rue Edouard Vaillant, Villejuif, F-94805 France

## Abstract

In genome editing with CRISPR–Cas9, transgene integration often remains challenging. Here, we present an approach for increasing the efficiency of transgene integration by homology-dependent repair (HDR). CtIP, a key protein in early steps of homologous recombination, is fused to Cas9 and stimulates transgene integration by HDR at the human *AAVS1* safe harbor locus. A minimal N-terminal fragment of CtIP, designated HE for HDR enhancer, is sufficient to stimulate HDR and this depends on CDK phosphorylation sites and the multimerization domain essential for CtIP activity in homologous recombination. HDR stimulation by Cas9–HE, however, depends on the guide RNA used, a limitation that may be overcome by testing multiple guides to the locus of interest. The Cas9–HE fusion is simple to use and allows obtaining twofold or more efficient transgene integration than that with Cas9 in several experimental systems, including human cell lines, iPS cells, and rat zygotes.

## Abstract (web summary)

The integration of exogenous DNA into the genome using CRISPR–Cas9 often presents a challenge to researchers. Here the authors fuse CtIP to Cas9 to stimulate recombination at target loci.

## Introduction

The CRISPR–Cas9 system has hugely facilitated genome editing. In pioneer studies with mammalian cells, the introduction of a DNA double-strand break (DSB) at a unique position in the genome, using the homing endonuclease I-SceI, allowed to stimulate gene targeting by homologous recombination^1,2^. Subsequently, different artificial sequence-specific nucleases, such as zinc finger and TALE nucleases, and more recently CRISPR–Cas9 nucleases have been used to target predetermined genomic sites^3–7^. With CRISPR–Cas9, a guide RNA with a spacer sequence complementary to the target DNA directs DNA cleavage by the Cas9 endonuclease^8^. Modification of the genome sequence takes place during DSB repair, and the molecular pathways that come into play determine the type of sequence change. Canonical nonhomologous end joining (cNHEJ) and alternative end-joining pathways such as micro-homology-mediated end joining (MMEJ) proceed by ligation of DNA ends after they have been processed and result in targeted but imprecise indels (generally small insertions or deletions). Microhomologies of two or more nucleotides may be exposed after DNA cleavage through resection and may be used during repair by MMEJ. In contrast to end-joining pathways, homology-dependent repair (HDR) using an exogenous DNA repair template supports precise genome editing. Typically, a transgene with homology arms to sequences flanking the DSB can be used and the transgene will thus be precisely integrated. In order to enhance genome editing by HDR, different strategies have been developed so far. For example, when cells are synchronized in S/G2 phases, the cell-cycle phases to which DNA repair by homologous recombination are restricted, and HDR can be increased up to fivefold^9^. NHEJ inhibition, for instance, following Ligase 4 inactivation, can also increase HDR^10^. Another approach has been to fuse the Geminin degron to Cas9 in order to induce its degradation in G1 and restrict target DNA cleavage to S/G2 phases^11,12^.

Here, we report a simple approach to increase HDR using Cas9 nuclease fused to an N-terminal domain of CtIP, a key protein in early steps of homologous recombination. This approach forces CtIP to the cleavage site and increases transgene integration by HDR. An N-terminal fragment of CtIP, called HE for HDR enhancer, is sufficient for HDR stimulation and requires the CtIP multimerization domain and CDK phosphorylation sites to be active. HDR stimulation with Cas9–HE resulted in an increase of twofold or more in the frequency of targeted transgene integration at independent loci tested in multiple systems, including human cell lines, human iPS cells, and rat zygotes. HDR stimulation by the Cas9–HE fusion depends on the guide RNA used, and this limitation may be overcome by testing multiple guide RNAs. In the absence of donor DNA, Cas9–HE induced a pattern of indels different from Cas9, and deletions between short stretches of homologous sequences were favored, suggesting that cNHEJ was partially inhibited and MMEJ was enhanced, likely due to the stimulation of DNA resection by the HE domain. Using Cas9–HE is straightforward, does not require using genetically modified cells or pharmacological reagents, and our results suggest that DNA repair pathways can be biased locally, at the site of DNA cleavage, to favor HDR and precise genome editing.

## Results

### Forcing CtIP to the DNA break site stimulates HDR

CtIP is a key protein in the initial step of homologous recombination that acts as a cofactor for MRE11 endonuclease in triggering DNA end resection^13–16^. In order to favor transgene integration by HDR, we sought to force CtIP recruitment to the DNA cleavage site by two different approaches based on fusion of CtIP to catalytically inactive Cas9 (dCas9) or to Cas9.

**Fig. 1:**
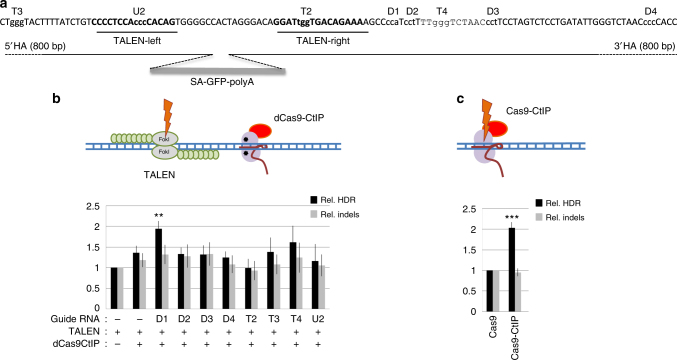
Forcing CtIP recruitment to the DNA cleavage site stimulates targeted transgene integration. **a** Distribution of TALEN and guide RNAs at *AAVS1* safe harbor locus. gRNAs are indicated on top of their corresponding PAM motif, which is shown as lowercase in the sequence. The donor DNA used had 5′ and 3′ homology arms as indicated. **b** Relative HDR and indel frequencies induced by TALEN and dCas9–CtIP recruitment near the cleavage site using different guide RNAs. Human RG37 fibroblasts were transfected with the indicated plasmids and GFP transgene donor with homology arms to the targeted *AAVS1* locus. HDR-mediated transgene integration was measured by FACS analysis of GFP-positive cells resulting from targeted GFP transgene integration. Indels at the cleavage site were measured by the T7E1 assay. The results are expressed as the mean of relative HDR or indel frequencies calculated by normalizing HDR or indel frequencies by that induced by TALEN transfection alone. Asterisks indicate the difference that is statistically significant when comparing cotransfection of dCas9–CtIP, guide RNA, and TALEN to TALEN-alone transfection in *t*-test (*P<0.05). Data are from three independent experiments. Error bars indicate standard deviation. **c** Relative HDR and indel frequencies induced by Cas9 and Cas9–CtIP at the cleavage site directed by T2 guide RNA. The results are expressed as the mean of relative HDR or indel frequencies calculated by normalizing HDR or indel frequencies by that induced by Cas9. Asterisks indicate that the difference is statistically significant when comparing Cas9–CtIP to Cas9 in *t*-test (**P<0.005). Data are from three independent experiments. Error bars indicate standard deviation. Guide RNA T2 was used because in contrast to other guides, it did not cleave the donor DNA available

We first targeted DNA cleavage at the *AAVS1* locus with TALENs and coexpressed dCas9–CtIP with guide RNAs specific to sequences next to the TALEN cleavage site (Fig. 1a) in RG37DR cells, human fibroblasts transformed by SV40^17^. *AAVS1* is a well-established model locus for genome-editing experiments in human cells and is generally considered to be a safe harbor locus for transgene integration^18^. This approach stimulated the integration of a GFP transgene with 800-bp homology arms by twofold with guide RNA D1, one of the guide RNAs lying closest to the TALEN cleavage site (Fig. 1b). Other guide RNAs did not efficiently stimulate the integration. This finding indicated that the expression of dCas9–CtIP is not sufficient, by itself, to stimulate integration and suggested that the position of dCas9–CtIP binding relative to the cleavage site is critical. The frequency of indels induced by *AAVS1* TALENs, measured by the T7 endonuclease I (T7EI) assay, was not significantly modified by co-expression of dCas9–CtIP (Fig. 1b).

We next tried to force CtIP to the DSB by direct fusion of CtIP to Cas9 nuclease. T2 guide RNA was used to induce cleavage next to the *AAVS1* TALEN cleavage site. The Cas9–CtIP fusion allowed to stimulate GFP cDNA integration by twofold compared to Cas9 (Fig. 1c). In contrast, the frequency of indels was not significantly modified when using Cas9–CtIP compared to Cas9 (Fig. 1c).

Altogether, these results show that forcing CtIP recruitment to the nuclease cleavage site, through fusion to either dCas9 or Cas9, can significantly stimulate targeted integration of a transgene.

### Identification of minimal HDR enhancer fragment of CtIP

**Fig. 2:**
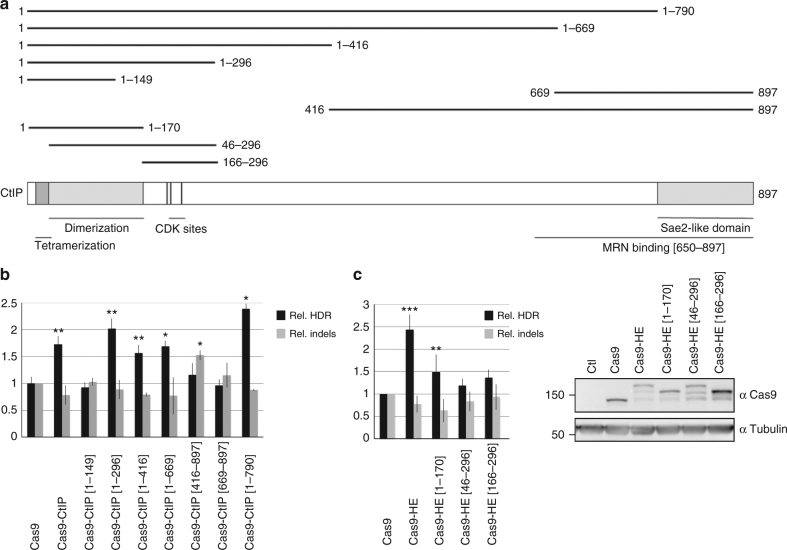
Identification of the “HDR-enhancer” (HE) domain of CtIP. **a** Schematic diagram of CtIP protein showing the different truncated CtIP proteins that have been fused to Cas9 and tested for their ability to stimulate HDR. Various sequence features of CtIP, including tetramerization and dimerization domains, and CDK phosphorylation sites S233, T245, and S276, are indicated. **b** Identification of a domain of CtIP, called HE, spanning aa 1 to 296, which is able to stimulate HDR when fused to Cas9. Human RG37 fibroblasts were transfected with the indicated plasmids expressing Cas9 or Cas9–CtIP derivatives, T2 guide RNA plasmid, and GFP transgene donor with homology arms to the targeted *AAVS1* locus. Expression of fusion proteins was examined by western blot (Supplementary Fig. 1). Data are from four independent experiments. Error bars indicate standard deviation. **c** Functional analysis of HE domain. HEK293 cells were transfected with the indicated Cas9 plasmids, T2 guide RNA, and GFP transgene donor with homology arms to the *AAVS1* targeted locus. HDR-mediated transgene integration was measured by FACS analysis of GFP-positive cells, resulting from targeted GFP transgene integration. Indels at the cleavage site were measured by the T7E1 assay. The results are expressed as the mean of relative HDR or indel frequencies calculated by normalizing every HDR or indel frequency by that induced by Cas9, respectively. Asterisks indicate that the difference is statistically significant when comparing Cas9–CtIP or Cas9–HE derivatives to Cas9 in nonparametric *t*-test (*P<0.05, **P<0.005, or ***P<0.0005). Data are from four independent experiments. Error bars indicate standard deviation. The relative expression levels of Cas9 and Cas9–HE derivatives were analyzed by western blot using anti-Cas9 and control anti-tubulin antibodies

In order to identify a minimal CtIP domain that can be used to stimulate homology-dependent insertion of a transgene, we tested the activity of two series of CtIP deletions, progressively removing fragments of ~200 amino acids (aa) starting from N- or C-terminal ends (Fig. 2a). Truncated CtIP proteins were fused to Cas9 nuclease and tested in RG37DR cells for GFP transgene integration at the *AAVS1* locus using T2 guide RNA. When C-terminal deletions were tested, we observed that deleting from aa 296 to the C-terminal end of CtIP did not affect HDR stimulation, and that the fragment from aa 1 to 296 was sufficient to stimulate HDR as efficiently as full-length CtIP (Fig. 2b). Conversely, when testing N-terminal deletions, we observed that deleting the fragment from aa 1 to 416 eliminated the HDR stimulation effect. The two N-terminal deletions tested were unable to stimulate HDR, although inducing roughly similar levels of imprecise mutations as measured by the T7EI assay (Fig. 2b). We conclude that the N-terminal fragment of CtIP from aa 1 to 296 is sufficient for HDR stimulation. The N-terminal fragment from aa 1 to 296 was coined as HE for HDR-Enhancer domain, and the corresponding Cas9 fusion was designated as Cas9–HE.

### Phosphorylation and multimerization domain essential to HE

Next, to examine how the small HE domain of CtIP stimulates transgene integration by HDR, we tested different Cas9–HE mutants in HEK293 cells. The HE domain includes several sequences recently reported to be essential for CtIP activity: the CtIP dimerization and tetramerization domains^19^, and three out of six CDK phosphorylation sites necessary for CtIP activity in HR^20^ (shown in Fig. 2a). First, we tried to dissect HE into smaller fragments, HE[1–170] that includes CtIP dimerization and tetramerization domains but lacks the three CDK phosphorylation sites, HE[46–296] lacking the first 45 aa of CtIP needed for interaction with the Mre11–Rad50–Nbs1 (MRN) complex^20^, and tetramerization^19^ and HE [166–296] containing the three CDK phosphorylation sites (shown in Fig. 2a). None of the HE fragments tested stimulated the integration of the GFP transgene, suggesting that all the different functional domains previously reported to be essential for CtIP activity are important for the full HE effect (Fig. 2c). Next, the three CDK sites were mutated either to alanine (Cas9–HE(3A)), to block phosphorylation, or to glutamic acid (Cas9–HE(3E)), to mimic phosphorylation by CDKs (Supplementary Fig. 2A). In HEK293 cells, the Cas9–HE(3E) mutant led to GFP transgene integration levels comparable to those achieved with Cas9–HE (Supplementary Fig. 2B). In contrast, when using the Cas9–HE(3A) mutant, which cannot be phosphorylated by CDKs, the levels of GFP transgene integration were similar to those achieved with Cas9, showing that the three CDK phosphorylation sites are essential for stimulating HDR with the HE domain. Finally, we wanted to test if the activity of the CtIP tetramerization domain is necessary. For that purpose, we used a L27E mutation shown to abrogate CtIP tetramerization while preserving dimerization^19^. We found that in HEK293 cells, it did not affect transgene integration compared to Cas9–HE (Supplementary Fig. 2C). The mutants were tested in a second cell line, HCT116 cells, and transgene integration was no longer stimulated with Cas9–HE(3A) nor with Cas9–HE(L27E), even though all proteins were expressed at similar levels as detected by western blot, pointing to a more important role of the tetramerization site in HCT116 cells (Supplementary Fig. 2D). The different impact of the L27E mutation in HEK293 and HCT116 cells suggests that the importance of CtIP tetramerization activity may vary depending on cell lines, perhaps due to the differences in the abundance of transfected proteins or endogenous CtIP. In any case, it therefore appears that the interplay of several functional features of CtIP, including known CDK phosphorylation sites and CtIP multimerization domain [1–170], is essential for the activity of the HE domain and that the HE domain as defined here is the smallest possible fragment that can be used to stimulate transgene integration.

### Cas9–HE induces a different pattern of indels than Cas9

Recent studies have indicated that the pattern of indels induced by Cas9 is not random and is determined by the spacer sequence rather than genomic context^21^. In addition, the mutation pattern could be modified by the DNA-PK inhibitor NU7441, which inhibits end-joining by cNHEJ, suggesting that the mutation pattern is dependent on the DNA repair pathways that have been involved. We were therefore interested in examining whether Cas9–HE induces a different pattern of indels than that of Cas9. Two guide RNAs, Spacer 54 and Spacer 93 targeting JAK and PCSK genes, respectively, which were previously characterized by van Overbeek et al.^21^ and the T2 guide RNA targeting the *AAVS1* locus were tested in HEK293 cells and the mutation pattern determined by deep sequencing of PCR products of the target loci. The proportion of mutant reads obtained with Cas9–HE and Cas9 was similar for Spacer 54 and Spacer 93, while for guide T2, Cas9–HE gave ~50% fewer mutant reads than Cas9. We examined in detail indels representing more than 2% of mutant reads for Cas9 and Cas9–HE. Depending on the guide RNA, they corresponded to seven to nine different indels that taken all together represented 47–70% of the total mutant reads. Interestingly, for all three guides, we observed that the patterns of indels induced by Cas9–HE were different from those induced by Cas9. The extent of changes, however, depended on the guide RNA (Table 1). As a control, we repeated the NU7441 treatment of Cas9-transfected cells that was previously reported^21^. Interestingly, for all three guide RNAs, Cas9–HE, and NU7441 treatment resulted for most indels in similar types of changes compared to those of Cas9 (changes were similar for 20 out of 24 indels). The differences, however, were generally of greater amplitude with NU7441. In particular, for spacer 54, the two most frequent mutations observed with Cas9 were reduced to tenfold by NU7441 treatment but only twofold when using Cas9–HE. This is reminiscent of the effects of lower NU7441 doses observed by van Overbeek et al.^21^. We also noted that indels with increased frequency were almost all deletions flanked by microhomologies. When comparing Cas9–HE to Cas9, 13 out of 14 indels with increased frequency were deletions flanked by microhomologies of two or more nucleotides and 10 out of 12 for NU7441 treatment. Taken together, these results are consistent with Cas9–HE inducing a different balance of end-joining pathways compared to those of Cas9 and having an effect similar to a low NU7441 dose, with a partial inhibition of cNHEJ and an increase of MMEJ, likely due to stimulation of resection by the HE domain.

**Table 1:** Indel mutation patterns induced by Cas9, Cas9–HE, and Cas9+NU7441

Indel mutation patterns induced after transfection of nucleases and guide RNA expression vectors were determined by sequencing of PCR amplicons of the targeted region. The wild-type target sequence is indicated on top of mutant sequences. When indicated, cells were treated with 10 μM DNA-PK inhibitor NU7441

The indels shown are indels that represented more than 2% of mutant reads obtained with Cas9 or Cas9–HE. If present, microhomologies (MH) of two or more nucleotides flanking the deletion are indicated

Spacer 54 and Spacer 93 are from guide RNAs previously analyzed by van Overbeek et al.^21^

For spacer 54, mutant reads were 35.7% (of the total 47,199 reads), 29.8% (of the total 48,265 reads), and 6.5% (of the total 116,354 reads) for Cas9, Cas9–HE, and Cas9+NU7441, respectively. For spacer 93, mutant reads were 31.3% (of the total 45,398 reads), 24.2% (of the total 55,573 reads), and 4.1% (of the total 36,979 reads) for Cas9, Cas9–HE, and Cas9+NU7441, respectively. For T2 guide RNA, mutant reads were 39% (of the total 68,852 reads), 16.8% (of the total 67,815 reads), and 31.8% (of the total 69,696 reads) for Cas9, Cas9–HE, and Cas9+NU7441, respectively

### Equivalent HDR stimulation by Cas9–HE and Cas9–HE–Geminin

**Fig. 3:**
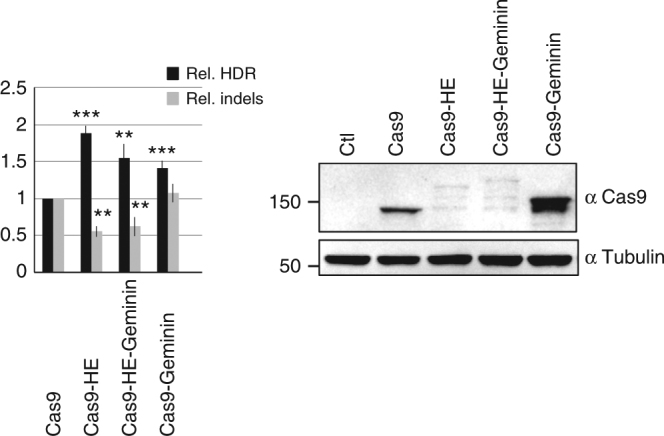
Stimulation of transgene integration by Cas9–HE and Cas9–Geminin. Relative frequencies of HDR and indels induced by Cas9 or fusion of Cas9 to HE domain, Geminin degron, or both. Human HEK293 cells were transfected with the indicated Cas9 plasmids, T2 guide RNA, and GFP transgene donor with homology arms to the *AAVS1* targeted locus. HDR-mediated transgene integration was measured by FACS analysis of GFP-positive cells, resulting from targeted GFP transgene integration. Indels at the cleavage site were measured by the T7E1 assay. The results are expressed as the mean of relative HDR or indel frequency calculated by normalizing every HDR or indel frequency by that induced by Cas9. Asterisks indicate that the difference is statistically significant when comparing Cas9–HE, Cas9–HE–Geminin, and Cas9–Geminin to Cas9 in *t*-test (*P<0.05 or **P<0.005). Data are from three independent experiments. Error bars indicate standard deviation. Relative expression levels of Cas9 and other fusions were analyzed by western blot with anti-Cas9 and control anti-tubulin antibodies. Protein extracts were obtained with lysis buffer containing 150 mM NaCl, which resulted in inefficient solubilization of Cas9 fusions with the HE domain compared to those of Cas9 and Cas9–Geminin

Fusion of the 110-aa degron of Geminin to Cas9 can increase homology-directed transgene integration^11^. The Geminin degron and HE domain of CtIP are expected to stimulate HDR by different mechanisms. In order to examine whether their activities could be additive, we compared Cas9–HE and Cas9–HE–Geminin fusions at the *AAVS1* locus in HEK293 cells. We therefore tested the activity of a Cas9–HE–Geminin fusion, containing both the HE domain and the Geminin degron. However, addition of the Geminin degron did not further enhance HDR compared with Cas9–HE (Fig. 3). One likely explanation is that HDR stimulation by Cas9–HE may already be limited to the G2/S phases of the cell cycle so that fusion to the Geminin degron and the resulting degradation during the G1 phase cannot further enhance the activity of Cas9–HE. The results obtained with Cas9–Geminin were similar to those reported by Gutschner et al., and the activity of Cas9–HE appeared slightly more efficient than that of Cas9–Geminin (Fig. 3).

### HDR stimulation in human iPS cells and rat oocytes

**Fig. 4:**
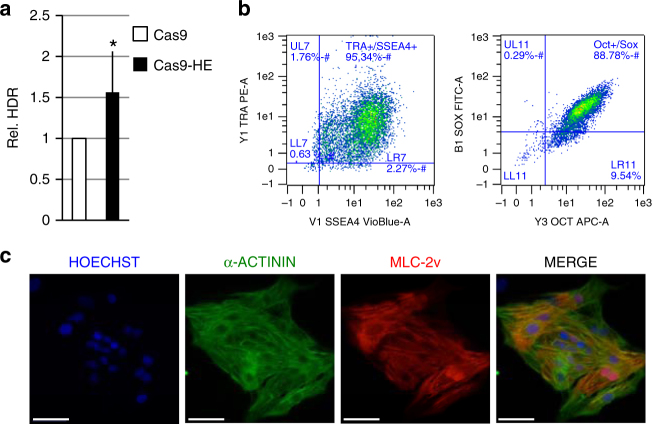
Stimulation of transgene integration by Cas9–HE in iPS cells. **a** Relative frequencies of HDR induced by Cas9–HE at DNA cleavage site directed by T2 guide RNA. Human iPS cells were transfected with the indicated Cas9 plasmids, T2 guide RNA, and puromycin transgene donor with 800-bp homology arms to the *AAVS1* locus. HDR-mediated transgene integration was measured by counting puromycin-resistant clones, resulting from targeted integration at the *AAVS1* locus of the puromycin resistance exon carried by the transgene. The results are expressed as the mean relative number of puromycin-resistant clones after normalizing the number of puromycin-resistant clones by that induced by Cas9. The difference between Cas9–HE and Cas9 was statistically significant (*t*-test, *P<0.05). Data represented are from four independent experiments. Error bars indicate standard deviation. **b** A clone of puromycin-resistant cells derived from puromycin selection of cells transfected with Cas9–HE, T2 guide RNA, and puromycin-resistant transgene donor was analyzed for expression of stemness markers TRA-1-60 and SSEA4 or Oct3/4 and Sox2 by FACS analysis. **c** After induction of cardiomyocyte differentiation in the clone analyzed in **b**, expression of cardiomyocyte markers alpha-actinin and MLC2-v was analyzed by immunohistochemistry. Scale bar, 50 µm

We next compared the efficiency of Cas9–HE and Cas9 for the targeted integration of transgenes in different experimental contexts. We first examined Cas9–HE activity in human iPS cells, an important model system for biomedical research. In preliminary experiments, we could not detect GFP-positive cells after cotransfection of the GFP transgene donor with Cas9 targeting the *AAVS1* locus. This was perhaps due to insufficient GFP expression from the *AAVS1* locus, and we therefore replaced the GFP cDNA with a puromycin resistance cDNA in the donor plasmid. After cotransfection of the puromycin resistance donor with T2 guide RNA and Cas9 or Cas9–HE expression vectors, puromycin selection was applied for 2 weeks and puromycin-resistant colonies were counted. As found above in immortalized RG37DR fibroblasts, HEK293, and HCT116 cells, Cas9–HE resulted in more efficient HDR than Cas9 in human iPS cells (Fig. 4a). Several clones of puromycin-resistant cells were further analyzed. At the DNA level, targeted integration of the puromycin cDNA cassette was validated by PCR amplification of the expected junctions between the donor construct and the target locus (Supplementary Fig. 3). Recent reports indicate that DSB repair pathways are important for cell reprogramming and properties of iPS cells^22,23^. We therefore wanted to examine whether Cas9–HE may have modified the behavior of iPS cells. Importantly, stem cell morphology of clones was similar for cells treated with either Cas9–HE or Cas9. Furthermore, in the clones tested, the expression of stemness markers was maintained (Fig. 4b), and cardiac differentiation could be efficiently induced (Fig. 4c), suggesting that treatment with Cas9–HE did not compromise the basic features of iPS cells. One potential concern with overexpression of Cas9–HE is that it might interfere with endogenous CtIP activity. In order to examine this possibility, we performed a RPA foci formation assay. After resection mediated by CtIP during DSB repair by HR, 3′ single-stranded DNA is initially bound by RPA and the formation of RPA foci is therefore a standard marker of DNA resection. Cells were transfected with Cas9–HE, Cas9–CtIP, or Cas9 as well as with siRNA directed toward CtIP or control. Two days after transfection, cells were X-ray irradiated to induce DSBs and RPA foci counted at 1, 2, 4, 6, and 8 h afterward (Supplementary Fig. 4). CtIP knockdown mildly decreased RPA foci formation (*p*<0.0005), while none of the Cas9 versions, i.e., Cas9, Cas9–CtIP, nor Cas9–HE significantly affected RPA foci formation. These results suggest that overexpression of Cas9–HE does not interfere with endogenous CtIP activity and does not seem to perturb the cell’s general ability to cope with DNA DSBs.

Next, we wanted to test the activity of Cas9–HE in rat oocytes. For this purpose, we co-microinjected into rat zygotes a long donor DNA (containing 4.7 kbp flanked by homology arms of ~1 kbp), sgRNAs targeting the *Rosa26* or *Il22bp* locus and Cas9–HE or Cas9 mRNA. As indicated in Table 2, zygotes that survived microinjection were reimplanted in foster mothers, and embryos at day 14 of gestation were harvested and genotyped (using the strategy depicted in Supplementary Fig. 5). For both *Rosa26* and *Il22bp* loci, sequencing of PCR amplicons spanning the targeted sequence revealed similar frequencies of indels, due to DSB repair by end-joining pathways, in Cas9–HE and Cas9 mRNA injections. In contrast, integration by HDR was strongly increased at the *Rosa26* locus in zygotes microinjected with Cas9–HE compared to those of Cas9 (representing 8% and 1% of harvested embryos for Cas9–HE and Cas9, respectively) but not at the *Il22bp* locus (representing 2% and 4% of harvested embryos for Cas9–HE and Cas9, respectively).

**Table 2:** Comparison of Cas9–HE and Cas9 in obtaining targeted transgene integration into the rat *Rosa26* and *Il22bp* loci

Cas9 form	Target locus	KI fragments	Dose Cas9 mRNA/sgRNA/donor DNA (ng/µl)	Eggs injected (survival rate %)	Eggs transferred	E14 embryos (% transferred)	Indels + (% of E14)	Donor integration+ (% of E14)	HDR+ (% of E14)
Cas9–HE	*Rosa26*	CAG-GFP	50/10/2	216 (75%)	154	37 (24%)	29 (78%)	5 (13%)	3 (8%)
Cas9	*Rosa26*	CAG-GFP	50/10/2	284 (77%)	211	84 (39%)	62 (73%)	2 (2%)	1 (1%)
Cas9–HE	*Il22bp*	Il22bp-2A-GFP	50/10/2	277 (78%)	217	82 (37%)	45 (55%)	7 (8%)	2 (2%)
Cas9	*Il22bp*	Il22bp-2A-GFP	50/10/2	286 (80%)	226	91 (40%)	55 (60%)	8 (8%)	4 (4%)

HDR+, HDR-mediated GFP transgene integration was defined as positive in E14 embryos for which both 5′ and 3′ in–out PCRs were detected using primers in the transgene and outside the homology arms (with PCR products confirmed by DNA sequencing, schematic of PCR primer positions in Supplementary Fig. 5); indels+, E14 embryos in which indels were detected by sequencing of PCR amplicons from the targeted genomic region; donor integration, E14 embryos in which PCR amplification of the transgene was positive

### HDR stimulation depends on the guide RNA

**Fig. 5:**
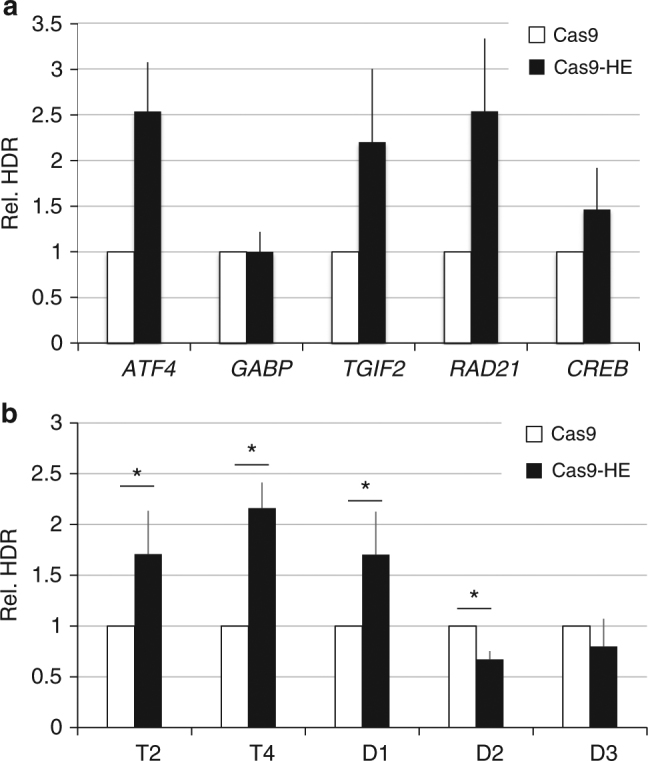
HDR stimulation by the HE domain takes place at different target genes and can depend on the guide RNA used. **a** Relative frequencies of HDR induced by Cas9–HE were compared to those induced by Cas9 at five different target genes in HEK293 cells using previously published guide RNAs and donor plasmids^36^. Targeted integration of the donor plasmid results in in-frame insertion of *E2A*-*neoR* cDNA^36^. G418(neomycin)-resistant colonies were counted after Cresyl violet staining to measure HDR-mediated events and normalized by the number of colonies obtained with Cas9 to give the relative HDR frequencies indicated. Data represented are from three independent experiments for *TGIF2*, *RAD21*, and *CREB* genes and from four experiments for *ATF4* and *GABP* genes and are provided in Supplementary Fig. 7. Error bars indicate standard deviation. **b** Relative frequencies of HDR induced by Cas9–HE were compared to those induced by Cas9 with the indicated guide RNAs, which all cleave to a small 50-bp region of the *AAVS1* locus, and a common p84∆ donor plasmid, harboring ~800-bp homology arms. P84∆ was derived from the p84 donor plasmid depicted in Fig. 1a by shortening the homology arms so that they would not be cleaved by any of the guide RNAs. Asterisks indicate that the difference is statistically significant when comparing Cas9–HE to Cas9 in *t*-test (*P<0.05). Data represented are from five independent experiments and are provided in Supplementary Fig. 7. Error bars indicate standard deviation

When experiments were performed in rats, transgene integration was increased at the *Rosa26* locus but not at the *Il22BP* locus. We therefore tested five additional target loci in human HEK293 cells and found that Cas9–HE stimulated more efficient transgene integration at four of the five sites tested (Fig. 5a). Several nonexclusive explanations could be considered to explain why integration was not stimulated at some targets, including a specific role of the target sequence or chromatin context. We wanted to examine the possibility that the guide RNA could play a role in determining whether Cas9–HE will stimulate HDR more efficiently than Cas9. We chose to compare five guide RNAs that all target cleavage in a short 50-bp sequence of the *AAVS1* locus. The homology arms in the donor DNA used in the experiments above (depicted in Fig. 1a) were first slightly shortened to avoid potential cleavage by the guide RNAs so that the same donor DNA could be used with all five guides. When Cas9–HE and Cas9 were compared with the different guides and modified donor, we found that Cas9–HE directed approximately twofold higher levels of transgene integration than Cas9 for guides T2, T4, and D1 but not with the other two guides tested, guides D2 and D3 (Fig. 5b). The results indicate that, unexpectedly, the stimulation of HDR by Cas9–HE is dependent on the guide RNA used to trigger genome editing. In order to further examine the role of the guide RNA sequence, we tested the impact of mutations of guide T4, the guide RNA which gave the strongest stimulation of HDR by Cas9–HE at the *AAVS1* locus. Out of 15 mutations tested, one mutation at position 3 of the guide RNA sequence eliminated the stimulation of HDR by Cas9–HE (mutant guide T4-m3, Supplementary Fig. 6A). This mutation decreased transgene integration and indel frequencies and therefore also interfered with DNA cleavage (Supplementary Fig. 6B and 6D, respectively). Interestingly, a second mutation, at position 18 of the guide RNA sequence, also significantly interfered with DNA cleavage (as shown from decreased indel and transgene integration frequencies for mutant guide T4-m18bis) but HDR could still be stimulated by Cas9–HE. PAM-distal and proximal sequences predominantly influence different steps of DNA cleavage^24^. Given the different effects of mutations at positions 3 and 18, we decided to test a truncated form of guide T4, lacking the first two ribonucleotides. HDR stimulation by Cas9–HE was no longer observed, while on the other hand, indel and transgene integration frequencies were not significantly affected. All together, these studies of mutant forms of guide T4 suggest that PAM-distal sequences may play a crucial role in determining the efficiency of HDR stimulation by Cas9–HE.

## Discussion

We describe here a simple approach to efficiently stimulate HDR after CRISPR–Cas9-mediated DNA cleavage. Our approach is based on forcing CtIP to the DNA cleavage site by using a Cas9–CtIP fusion. Such a fusion strategy was previously used to modulate DNA repair at the cut site, allowing to inhibit base-excision repair when fusing Cas9 to the uracil DNA glycosylase inhibitor from *Bacillus* *subtilis* bacteriophage^25^ or to increase indels when fusing Cas9 to *E. coli* Rec A^26^. This report shows that it can also be used to stimulate HDR. Previous approaches for stimulating HDR were based on cNHEJ inhibition or Rad51 recombinase stimulation by genetic or pharmacological manipulation of cells^10,27^. The direct fusion strategy reported here is based on targeted activity of a key component of the homologous recombination DNA repair pathway and is potentially less likely to have a global effect on DNA repair. Importantly, we show that HDR stimulation is due to the N-terminal 296 aa of CtIP (designated as the HDR-enhancer domain, HE). Activity of the HE domain of CtIP required several molecular features known to be essential to CtIP activity in homologous recombination. We speculate that the HE domain, normally involved in multimerization of CtIP, could recruit endogenous CtIP at the cleavage site, and thereby stimulate HDR with donor DNA.

As shown in Supplementary Fig. 2B three known CDK phosphorylation sites (S233, T245, and S276) are essential for HDR stimulation by the HE domain. These CDK sites are known to be required for the interaction of CtIP with Nbs1^20^. This finding suggests that Nbs1, and perhaps the whole Mre11–Rad50–Nbs1 endonuclease complex, may be recruited at the target site by Cas9–HE and may stimulate HDR.

During homologous recombination, CtIP and the MRN complex trigger end resection at the DSB, generating single-stranded DNA needed to search for and copy a DNA repair template. CtIP is also known to contribute to alternative end-joining, which requires resection and is mechanistically different from cNHEJ. Similarly, Cas9–HE may stimulate DSB repair by HR, as suggested by elevated transgene integration, as well as favor alternative end-joining pathways. Indeed, the mutation patterns were different for Cas9–HE and Cas9, suggesting that the balance of cNHEJ and MMEJ end-joining pathways is affected by the fusion of the HE domain to Cas9. The effect of Cas9–HE was reminiscent of the effects of low NU7441 dose reported by van Overbeek et al.^21^, suggesting that the HE domain may exert a mild inhibition of cNHEJ. In addition, deletions flanked by microhomologies had increased the frequency with Cas9–HE (Table 1), suggesting that MMEJ was favored relative to cNHEJ. These findings are consistent with the known role of CtIP in triggering DNA resection and antagonizing cNHEJ at the earlier steps of choice between the DSB repair pathways. The increased role of MMEJ may explain why, even though transgene integration is stimulated, the frequency of indels is not significantly different from Cas9–HE compared to those of Cas9.

Enhancement of HDR with Cas9–HE relative to Cas9 was observed at different loci, but unexpectedly, it was only observed for certain guides at a given locus (Fig. 5). One way to overcome this limitation may be to use several guides simultaneously. Alternatively, a side-by-side comparison of Cas9 and Cas9–HE may be performed for each candidate guide. The reason for such dependence on the guide RNA is not clear. Guide RNA sequence, however, is known to have different roles in Cas9 activity. For example, mismatches in the PAM-distal sequence can specifically inhibit conformational changes needed to activate Cas9 nuclease domains without affecting DNA binding^28^. A recent study also showed that association and dissociation kinetics of dCas9 to DNA are influenced by different parts of the guide RNA sequence, with dissociation being influenced by PAM-distal sequences^24^. Our finding that mutants of guide T4 with a PAM-distal mutation or with a deletion of the two most PAM-distal nucleotides can no longer support stimulation of integration by Cas9–HE that is reminiscent of these findings. One possibility is that in cells, specific guide RNA features such as PAM-distal sequences, influence how long Cas9–HE remains bound to DNA after cleavage and thereby impacts the activity of the HE domain on subsequent DNA repair. We propose that such features will also influence the efficiency of other Cas9 fusions.

As shown in the present work, using the Cas9–HE fusion allows to achieve more efficient HDR-mediated transgene integration at the *AAVS1* locus, which given its safe harbor properties, is an important locus for transgene integration in gene therapy and functional studies. Cas9–HE activity was demonstrated at the *AAVS1* locus in human-immortalized and transformed cell lines, as well as in iPS cells. Importantly, iPS features did not appear to be compromised during genome editing with Cas9–HE since modified clones expressed stemness markers and had preserved their ability to differentiate into cardiomyocytes. In addition, when the *Rosa26* locus was targeted in rat zygotes, injections with Cas9–HE mRNA led to higher frequency of transgene integration than Cas9 mRNA, showing that enhanced frequency of targeted integration can also be induced at another important safe harbor locus and that using Cas9–HE does not compromise development. The addition of Cas9–HE to the genome-editing toolbox opens a promising avenue for higher efficiency of precise genome editing with the CRISPR–Cas9 system. Further studies will aim at better understanding the importance of the guide RNA used and at combining additional regulatory domains of DNA repair proteins to further stimulate HDR. In particular, by further Cas9 engineering, it may be possible to overcome the inhibition of homologous recombination during the G1 phase, as suggested by recent studies of DNA DSB repair during the cell cycle^29^, and achieve efficient precision genome editing in quiescent cells.

## Methods

### Plasmids

TALE nucleases were produced starting with plasmids for TALE repeats kindly provided by Dr. Bo Zhang^5^. TALEN DNA-binding domains targeted the following sequences: CCCCTCCACCCCACAG (*AAVS1* TALEN-left) and TTTCTGTCACCAATCC (*AAVS1* TALEN-right). Guide RNA sequences were cloned in MLM3636-derived vector (Addgene #43860, a kind gift of the Joung lab), and Cas9-expression vector Addgene #41815 from the Church lab was used. CtIP-expression vector was kindly sent by Dr. Xiao Wu^20^. CtIP fragments were amplified by PCR and inserted between EcoRI and AgeI restriction sites in Cas9-expression vector by standard cloning, resulting in the fusion of CtIP fragments at the C-terminus of Cas9. GFP donor plasmid, containing a GFP transgene with an artificial splice acceptor site, *E2A-GFP* coding sequence, and bGH polyA sequence flanked by 800-bp homology arms to the *AAVS1* locus, was kindly provided by Dr. Urnov^18^. Guide RNAs and donor plasmids targeting the human *ATF4*, *GABP*, *TGIF2*, *RAD21*, and* CREB* genes were from the Mendenhall lab (Addgene #72350, #72351, #64253, and #64254).

### Cell culture and transfection

All cells were cultured at 37 °C in a humidified chamber with 5% CO_2_ and transfected with the AMAXA electroporation system. HEK293 cells were cultured in DMEM supplemented with 10% fetal bovine serum (FBS). iPSCs purchased from Univercell-Biosolutions were maintained on mTeSR1 basal medium supplemented with mTeSR1 5× Supplement (Stemcell Technologies). A total of 10^6^ HEK293 cells were nucleofected with 1 μg of Cas9 expression plasmid, 1 μg of gRNA expression plasmid, and 1 μg of GFP donor using V solution (Lonza) and Amaxa A-023 program. RG37 cells were cultured in DMEM supplemented with 10% FBS and nucleofected with 1 μg of Cas9 expression plasmid, 1 μg of guide RNA expression plasmid, and 1 μg of GFP donor using NHDF solution (Lonza) and Amaxa P-022 program. HCT116 cells were cultured in McCoy supplemented with 10% FBS and transfected with 4 μg of Cas9 expression plasmid, 2 μg of guide RNA expression plasmid, and 6 μg of GFP donor using V solution (Lonza) and Amaxa D-032 program. For experiments reported in Fig. 5b and Supplementary Fig. 6 and 7, HEK293 cells were transfected with Lipofectamine 3000 reagent (Thermo Fisher Scientific) according to supplier recommendations: 2 μg of Cas9 expression plasmid, 1 μg of guide RNA expression plasmid, and 1 μg of GFP donor were mixed with Lipofectamine 3000 and added to the culture medium of a well from a six-well plate that had been seeded the previous day with 0.7 × 10^6^ cells. Electroporation of iPS cells was performed according to the manufacturer’s instructions with Lonza 4D-Nucleofector System and the following parameters P3 Primary Cell 4D-Nucleofector program: CM-113. Differentiation of iPS cells into cardiomyocytes was performed according to a protocol adapted from ref. ^30^. The basal medium made with RPMI 1640 medium (ref. 11875-093, Life Technologies), 500 μg/ml human albumin (ref. A9731, Sigma), and 213 μg/ml l-ascorbic acid (ref. A8960, Sigma), was complemented with 4 μM CHIR99021 (ref. 77052, Stemcell Technologies) and 10 ng/ml BMP4 (ref.130-111-164, Miltenyi) from day 0 to day 2, or 2 μM Wnt C59 (ref. S7037, Selleckchem) from day 3 to day 4. From day 5, the cells were maintained in the basal medium. Beating areas were observed from day 9. HEK293 and HCT116 cells were purchased from ATCC, and RG37 is a human cell line derived from the SV40-transformed human fibroblasts GM639 by the laboratory of BL^31^. The parental GM639 cell line was obtained from Dr. Monnat RJ (University of Washington)^32^.

### Analysis of GFP transgene integration by FACS

When targeting the *AAVS1* locus with the GFP donor^18^, targeted integration of GFP cDNA results in cells becoming GFP-positive, which can be easily monitored by FACS analysis. Cells were analyzed for GFP expression by flow cytometry using an Accuri C6 analyzer (BD Biosciences) 6–7 days after transfection. The relative HDR frequency was calculated by normalizing HDR frequencies by the HDR frequency induced by Cas9 alone. GFP-positive cells ranged between 0.5% and 15% of cells analyzed depending on the cell line used and the experiment.

### Analysis of imprecise mutation frequencies by the T7EI assay

T7 endonuclease I (T7EI) assays were performed to analyze the frequency of imprecise mutations induced by end- joining pathways of DSB repair. Seventy-two hours after transfection, genomic DNA was extracted with DNA mini kit (EZNA tissue DNA kit, Omega Bioteck), and the AAVS1 target locus was amplified by PCR using the following primers: T7AAVFw 5′-cagcaccaggatcagtgaaa-3′ and T7AAVRev 5′-ctatgtccacttcaggacagca-3′. PCR products were denatured, annealed to form heteroduplexes in a PCR machine (5 min at 95 °C, 95–25 °C at −0.5 °C/30 s, and 15 min at 4 °C), digested with 1.5 units of T7E1 (New England Biolabs) for 10 min at 37 °C, and run on a 2.5% agarose gel. After migration, sequence modification frequencies were estimated as commonly done^33^: %indels =1–(1–Xc)^1/2^ with Xc, amount of cleaved products, and if Xc<0.15, %indels=Xc/2. %indels calculated from the T7E1 assay at the *AAVS1* locus varied between 2% and 20% depending on the cell line used and the experiment.

### Analysis of protein expression levels by western blot

Proteins were isolated 48 h after transfection. Cells were resuspended in lysis buffer (Tris-HCl 50 mM, pH 7, NaCl 150 mM or KCl 300 mM, Triton-X-100 1%, SDS O 1%, EDTA 1 mM, DTT 1 mM, aprotinin 1 μg/μl, pepstatin 10 μg/μl, and leupeptin 1 μg/μl), centrifuged at 15,000 × *g* for 15 min at 4 °C, and supernatants were used. We observed that lysis with buffer containing 300 mM KCl, which is known to favor the release of chromatin-bound proteins, allowed better extraction of Cas9–HE and derivatives compared to lysis buffer with 150 mM NaCl. Western blots were performed by standard Tris-glycine SDS-PAGE followed by transfer to nitrocellulose membranes. Following blocking with 5% BSA in TBS-T (Tris 24 mM, NaCl 137 mM, KCL 2.68 mM, and Tween-20 0.1%), membranes were probed with anti-Cas9 antibody (Novus Biologicals, NBP2-36440SS) at 1 μg/ml and anti-tubulin antibody (Sigma, T6074200UL) at 0.1 μg/ml, and visualized by chemiluminescence with a GBox (Syngene) or exposure to the X-ray film. Uncropped scans are provided as Supplementary Fig. 8.

### Analysis of indel mutation patterns

DNA was isolated from transfected cells (EZNA tissue DNA kit, Omega Bioteck), and the target region was amplified by PCR with Phusion Polymerase (NEB). Each sample was assigned to a primer set with a unique barcode to enable multiplex sequencing. PCR products were purified on a 2% agarose gel and treated by the MNHN genomics center and sequences on Ion Torrent PGM. A custom python pipeline was used to count and characterize indels as detailed in ref. ^31^. All sequence data from Table 1 are available from NCBI BioPRoject with the accession number PRJNA433647.

### Generation of genome-edited rats

Zygotes were obtained from superovulated Sprague-Dawley (SD/Crl) rats (Charles River, L'Arbresle, France) and microinjected as follows. Prepubescent females (4–5-weeks old) were superovulated with pregnant mare serum gonadotropin (30 IU; Centravet, France) and followed 48 h later with human chorionic gonadotropin (20 IU; Centravet, France) before breeding. Briefly, linearized excised donor DNA consisted of (i) when targeting the *Rosa26* locus, the CAG promoter controlling GFP expression flanked by homology arms of 800 bp of *Rosa26* contiguous to the site of cleavage directed by guide RNA with the following target sequence GTGTATGAAACTAATCTGTC TGG^34^ or (ii) when targeting the *IL22BP* locus, *E2A-GFP* cDNA flanked by 2-kbp homology arms to the following guide RNA target site TGTGTGCAGATTCCATAAAC TGG. The Cas9–HE or Cas9 mRNAs, sgRNA, and donor DNA were mixed (50, 10, and 2 ng/μl, respectively) and microinjected into the pronucleus and cytoplasm of zygotes. Zygotes surviving microinjection were implanted into pseudopregnant females. At day 14, females were sacrificed, and DNA was extracted from embryos for genotyping. Genotyping was performed using the primers and PCR conditions described in Supplementary Fig. 5 and Supplementary Table 1. PCR products were analyzed by microfluidic capillary electrophoresis^35^ and Sanger sequencing. The study was approved by the Ethics Committee on Animal Experimentation of the Pays de la Loire Region, France, in accordance with the guidelines from the French National Research Council for the Care and Use of Laboratory Animals (Permit Numbers: Apafis 692).

### Immunohistochemistry

Briefly, cells were fixed with PBS containing 8% paraformaldehyde for 20 min at 4 °C. After washing with PBS, cells were permeabilized and blocked with PBS containing 0.1% Triton-X-100 for 15 min at 4 °C. After washing with PBS, the cells were blocked with PBS containing 1% BSA and 10% horse serum for 1 h at room temperature. Then, the cells were incubated, with mouse anti-α-actinin antibody (dilution: 1/800; A7811; Sigma) and with rabbit anti-MYL2 antibody (dilution:1/600; 10906-1-AP; Proteintech), overnight at 4 °C. The cells were incubated the next day with goat anti-mouse IgG antibody conjugated to Alexa Fluor 488 (dilution:1/500; A-11001; Invitrogen) and a goat anti-rabbit IgG antibody conjugated to Alexa Fluor 555 (dilution:1/500; A-21428; Invitrogen) for 1 h at room temperature in the dark. Counterstaining was performed using Hoechst (dilution:1/4000; H3570, Invitrogen) for 10 min at room temperature. The stained cells were analyzed with a Nikon Eclipse Ti microscope.

### Flow cytometric analysis of iPS cells

iPSCs were singularized with Accutase. For the surface pluripotent markers, cells were resuspended in staining buffer. Then, the cells were incubated with monoclonal anti-SSEA-4 antibodies conjugated to Vioblue (dilution: 1/10; clone REA101, reference no. 130-098-366, MACS Miltenyi Biotec) and monoclonal anti-TRA-1-60 antibodies conjugated to PE (dilution:1/10; clone REA157, reference no. 130-100-350, MACS Miltenyi Biotec) at 4 °C for 10 min Finally, the cells were washed with stain buffer, centrifuged, and resuspended in a suitable amount of stain buffer for flow cytometry analysis. For the intracellular pluripotent markers, the cells were fixed using Cytofix for 20 min at 4 °C and washed with stain buffer. Then, the cells were incubated with monoclonal anti-OCT3/4 antibodies conjugated to APC (dilution:1/10; clone REA338, reference no. 130-105-607, MACS Miltenyi Biotec) and monoclonal anti-Sox2 antibodies conjugated to FITC (dilution:1/10; clone REA320, reference no. 130-104-993, MACS Miltenyi Biotec), and diluted in Perm/Wash buffer, at 4 °C for 30 min. Finally, the cells were washed with stain buffer, centrifuged, and resuspended in a suitable amount of stain buffer for flow cytometry analysis. For each sample, the events were captured on the MACSQuant VYB flow cytometer and analyzed with MACSQuantify Software.

### RPA foci formation assay

Twenty-four hours after plating, RG37 fibroblast cells were transfected with siRNA using Interferin (Polyplus, Ozyme). siNT(control): AUGAACGUGAAUUGCUCAA(dTdT). siCtIP: GCUAAAACAGGAACGAAUC. Three days after plating, cells were transfected with expression plasmids for Cas9, Cas9–HE, and Cas9-CtIP using JetPei (Polyplus, Ozyme). Five days after plating, cells were irradiated with X-rays at 6 Gy (XRAD 320, 1.03 Gy/min). At 0, 1, 2, 4, 6, and 8 h after irradiation, cells on coverslips were pre-permeabilized with PBS–Triton 0.25% for 3 min on ice, and then fixed in paraformaldehyde 2% for 15 min. The cells were then incubated with PBS containing 0.5% Triton-X-100 for 5 min at RT for permeabilization. After blocking in PBS containing 3% BSA and 0.05% Tween-20 solution for 30 min at RT, immunostaining was performed using the following primary antibody: mouse anti-RPA (1:300, ANA19L, Millipore). Incubation was performed for 1 h 30 min at 37 °C with antibodies diluted in PBS containing 3% BSA and 0.05% Tween-20. Next, the coverslips were incubated for 45 min with Alexa-488-conjugated anti-mouse secondary antibody (Life Technologies) at 37 °C and mounted in mounting medium (Dako) supplemented with 40,60-diamidino-2-phenylindole (DAPI) (Sigma). Images were captured using a Zeiss Axio Imager Z1 microscope with a ×63 objective equipped with a Hamamatsu camera. Acquisition was performed using AxioVision (4.7.2). Images were imported, processed, and merged in the ImageJ software.

### Statistical tests

Nonparametric Mann–Whitney t-tests were performed to determine statistically significant differences in transgene integration efficiency, and indels between different experimental conditions or the number of RPA foci per cell (*P<0.05, **P<0.005, and ***P<0.0005). Error bars indicate standard deviation.

### Data availability

The data generated in this study are available in the manuscript and accompanying documents or from the corresponding author upon reasonable request. Sequence data were submitted to NCBI and are accessible with BioProject ID: PRJNA433647.

## Electronic supplementary material

Supplementary Information(PDF 1923 kb)
