# Supplementary material for: CtIP fusion to Cas9 enhances transgene integration by homology-dependent repair
Source: Nat Commun. 2018 Mar 19;9:1133. doi: 10.1038/s41467-018-03475-7 (PMC5859065; doi:10.1038/s41467-018-03475-7)
Supplement: Supplementary file 1 — Supplementary Information(PDF 1923 kb) [file 41467_2018_3475_MOESM1_ESM.pdf]

Supplementary information

CtIP fusion to Cas9 enhances transgene integration by homology-dependent repair. Charpentier et al.

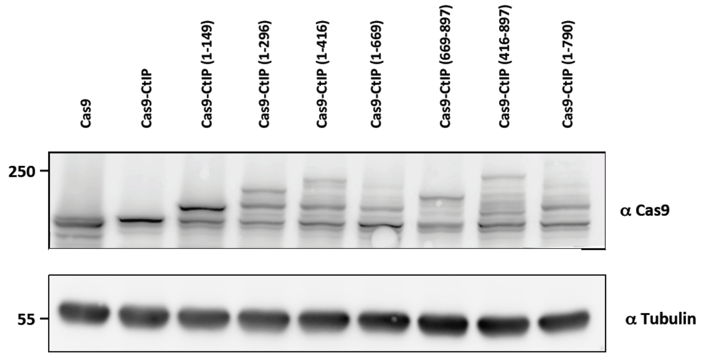

Supplementary Figure 1 Relative expression levels of Cas9 and Cas9-CtIP derivatives

Relative expression levels of Cas9 and Cas9-CtIP derivatives were analyzed by western blot with anti-Cas9 and control anti-tubulin antibodies.
Several fusion proteins of Cas9 and CtIP fragments (Cas9-CtIP, Cas9-CtIP[1-669] and Cas9-CtIP[1-790]) were not detected as bands of the expected size and only bands likely corresponding to degradation products could be detected. This may be due to unstability of the fusion proteins during extraction. Reminiscent of these difficulties, we note that in structure-function studies, CtIP was found to be very difficult to purify from insect cells with the baculovirus system (Davies et al., 2015) . We have also produced recombinant Cas9[1-296] in E Coli and found that solubilization and purification of full-length protein was very inefficient and that a product corresponding roughly to the Cas9 moiety was predominantly isolated.

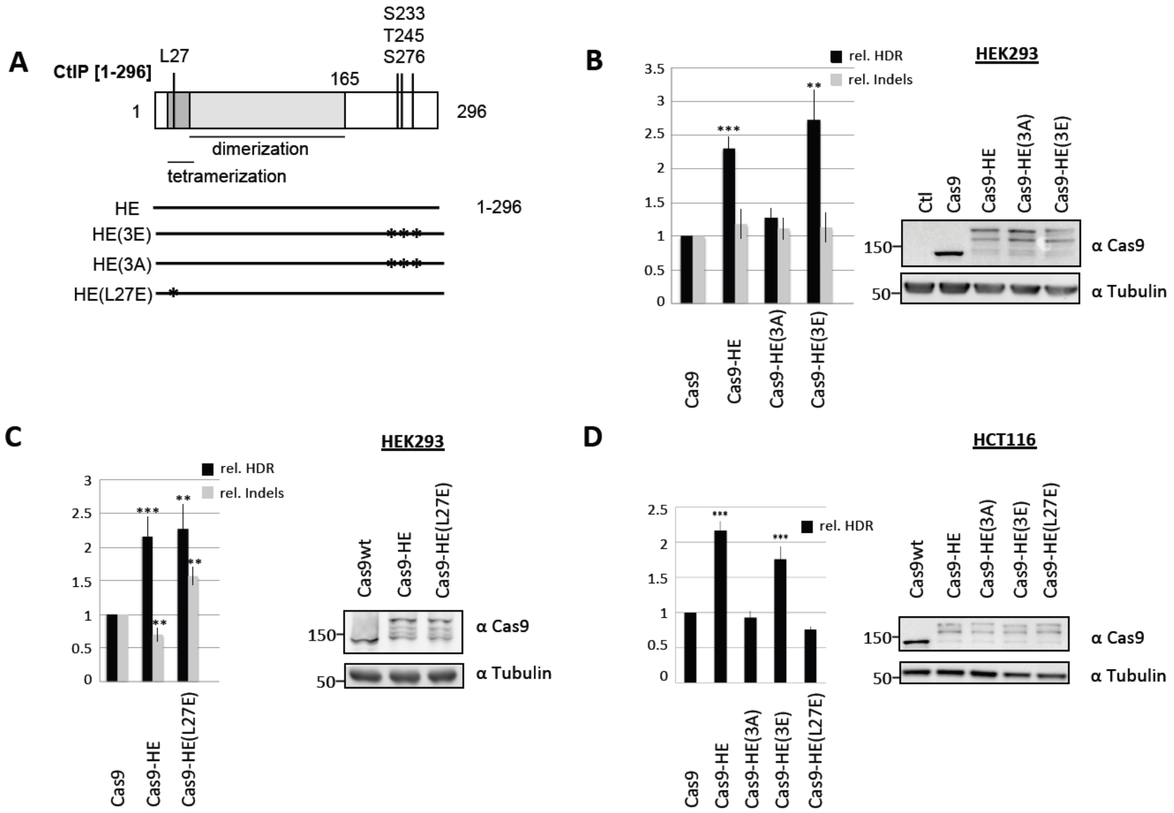

Supplementary Figure 2 Functional analysis of the HE domain

A) Schematic diagram of the HE domain, spanning aa 1 to 296 of CtIP, showing the different mutants that have been tested for their ability to stimulate HDR. Various sequence features of CtIP, including tetramerization and dimerization domains, L27 needed for tetramerization and CDK phosphorylation sites S233, T245 and S276, are indicated.
B) CDK phosphorylation sites are essential for HDR stimulation by the HE domain in HEK293 cells.
C) L27E mutation does not significantly impair activity of the HE domain in HEK293 cells.
D) CDK phosphorylation and L27 are essential for HDR stimulation by the HE domain in HCT116 cells.
Human HEK293 or HCT116 cells were transfected with the indicated plasmids expressing Cas9 or Cas9-HE derivatives, T2 guide RNA plasmid and GFP transgene donor with homology arms to the targeted AAVS1 locus. HDR-mediated transgene integration was measured by FACS analysis of GFP-positive cells resulting from targeted GFP transgene integration. Indels at the cleavage site were measured by the T7E1 assay. Results are expressed as mean of relative HDR or indels frequencies calculated by normalizing every HDR or indels frequency by that induced by Cas9 respectively. Asterisks indicate that difference is statistically significant when comparing Cas9-CtIP or Cas9-HE derivatives to Cas9 in non-parametric t-test (*, P<0.05, **, P<0.005 or ***, P<0.0005). Data in B, C and D are each from 4 independent experiments. Relative expression levels of Cas9 and Cas9-HE derivatives were analyzed by western blot using anti-Cas9 and control anti-tubulin antibodies.

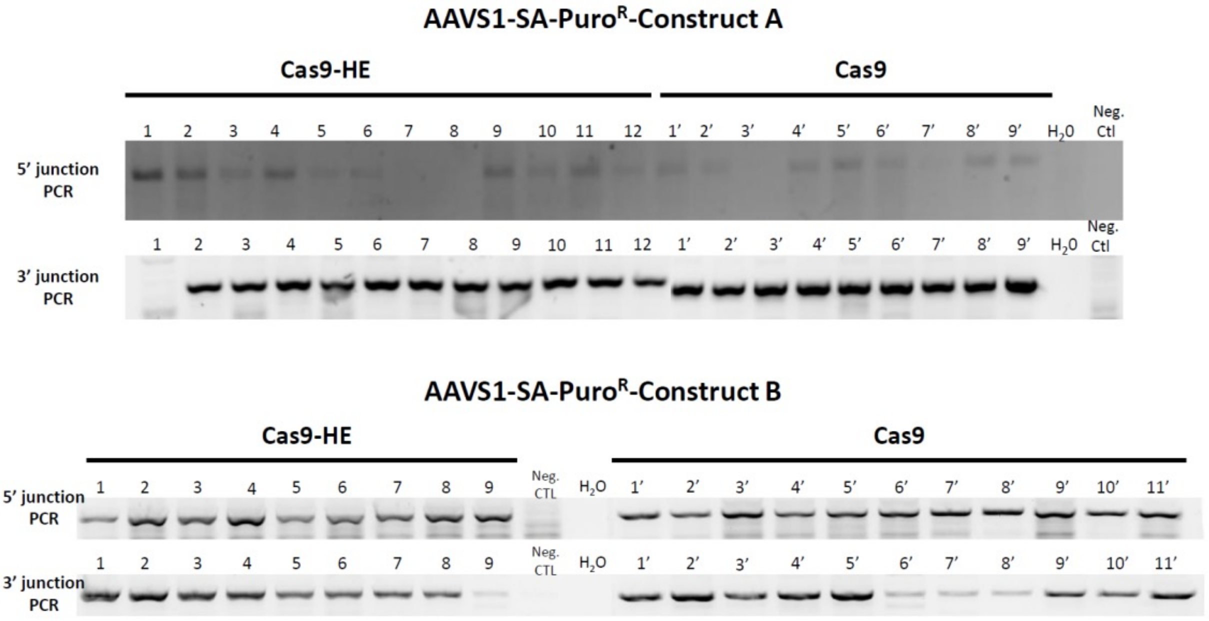

Supplementary Figure 3. Genome editing in iPS cells at the AAVS1 locus with Cas9-HE vs Cas9.

Human iPS cells were transfected with Cas9-HE, T2 guide RNA and puromycin-resistance transgene donors A (used in Figure 4) or B. After selection with puromycin, resistant colonies were expanded and analyzed for targeted integration by PCR analysis. The integration of the transgene was detected by junction PCR. Note, however, that Southern blotting would be needed to potentially reveal complex transgene integration patterns.

The two donor constructs were derived from the GFP donor plasmid used in Figures 1 to 3, replacing the SA-GFP-polyA cassette from (DeKelver et al., 2010) with a SA-Puro^R-polyA cassette and adding an independent transgene, A or B with a fluorescent Cherry reporter driven by a tissue-specific promoter.

A) For construct A, targeted integration of the transgene gives rise to a 931bp band for the 5' junction and to 890bp band for the 3' junction. 75% of the clones edited with Cas9-HE were correctly engineered while 89% of those generated with Cas9 were positive for targeted transgene integration.

B) For construct B, targeted integration of the transgene gives rise to a 931bp band for the 5' extremity and to 887bp band for the 3' end. 100% of the clones edited with Cas9-HE or with Cas9 were positive for targeted integration of the transgene.

A.

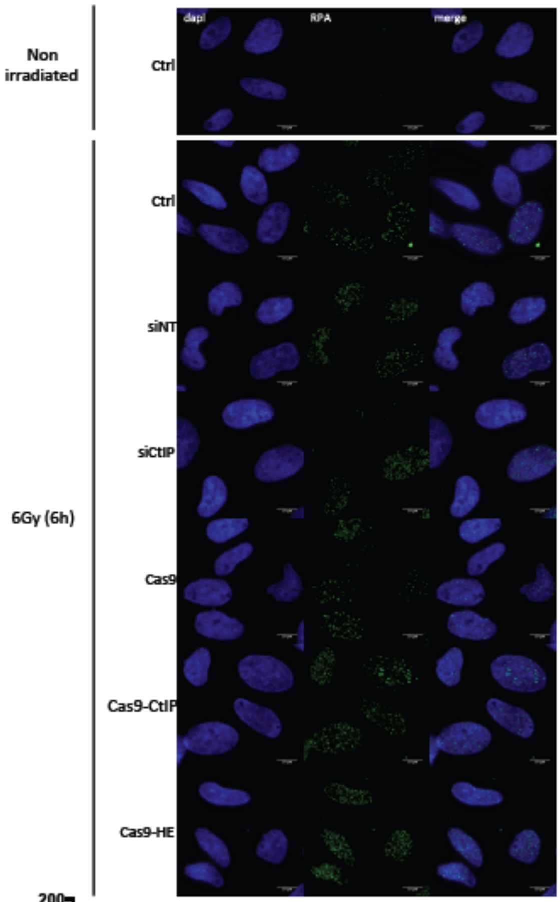

Non irradiated
Ctrl
dapi
RPA
merge
6Gy (6h)
Ctrl
siNT
siCtIP
Cas9
Cas9-CtIP
Cas9-HE

B.

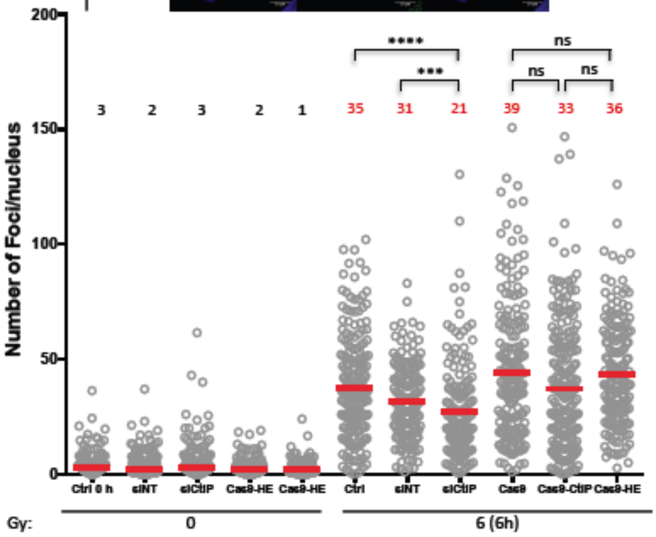

Number of Foci/nucleus
200
150
100
50
0
****
***
ns
ns
ns
3
2
3
2
1
35
31
21
39
33
36
Ctrl 0 h
siNT
siCtIP
Cas9-HE
Cas9-HE
Ctrl
siNT
siCtIP
Cas9
Cas9-CtIP
Cas9-HE
Gy:
0
6 (6h)

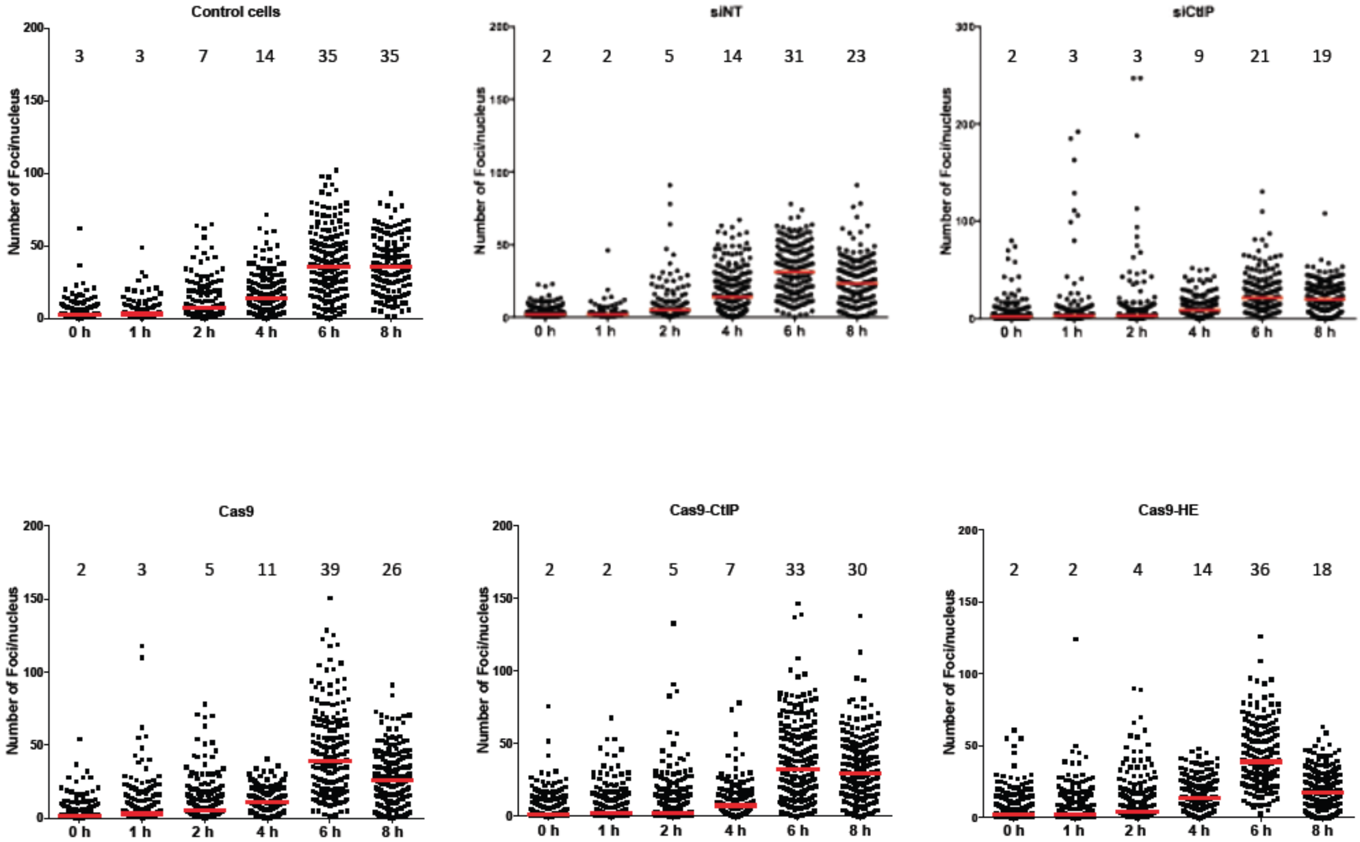

Supplementary Figure 4. RPA foci formation after X-ray irradiation

RPA foci were counted in control cells and at different times after X-ray irradiation in RG37 cells transfected with the indicated Cas9 fusions or anti-CtIP siRNA or control siRNA. Counts of RPA foci per nucleus are cumulated from three independent transfection experiments.

A) Detection of RPA foci by immunostaining with anti-RPA antibody (green) and counterstaining with DAPI (blue).

B)) Counts of RPA foci per nucleus are shown at 6 hours after irradiation, which corresponds to the peak of RPA foci per nucleus after irradiation (see full kinetics data in section C). Median number of foci per nucleus is indicated as a red bar, the values on the graph. Silencing CtIP expression diminished RPA foci number per cell compared to control cells and cells transfected with control siRNA (***, $p<0.0005$; ****, $p<0.0001$, nonparametric Mann-Whitney t-test) as expected while no difference was found between cells with Cas9, Cas9-CtIP or Cas9-HE.

C) Counts of RPA foci per nucleus are shown at the indicated times after irradiation. Median number of foci per nucleus is indicated as a red bar, the values on the graph.

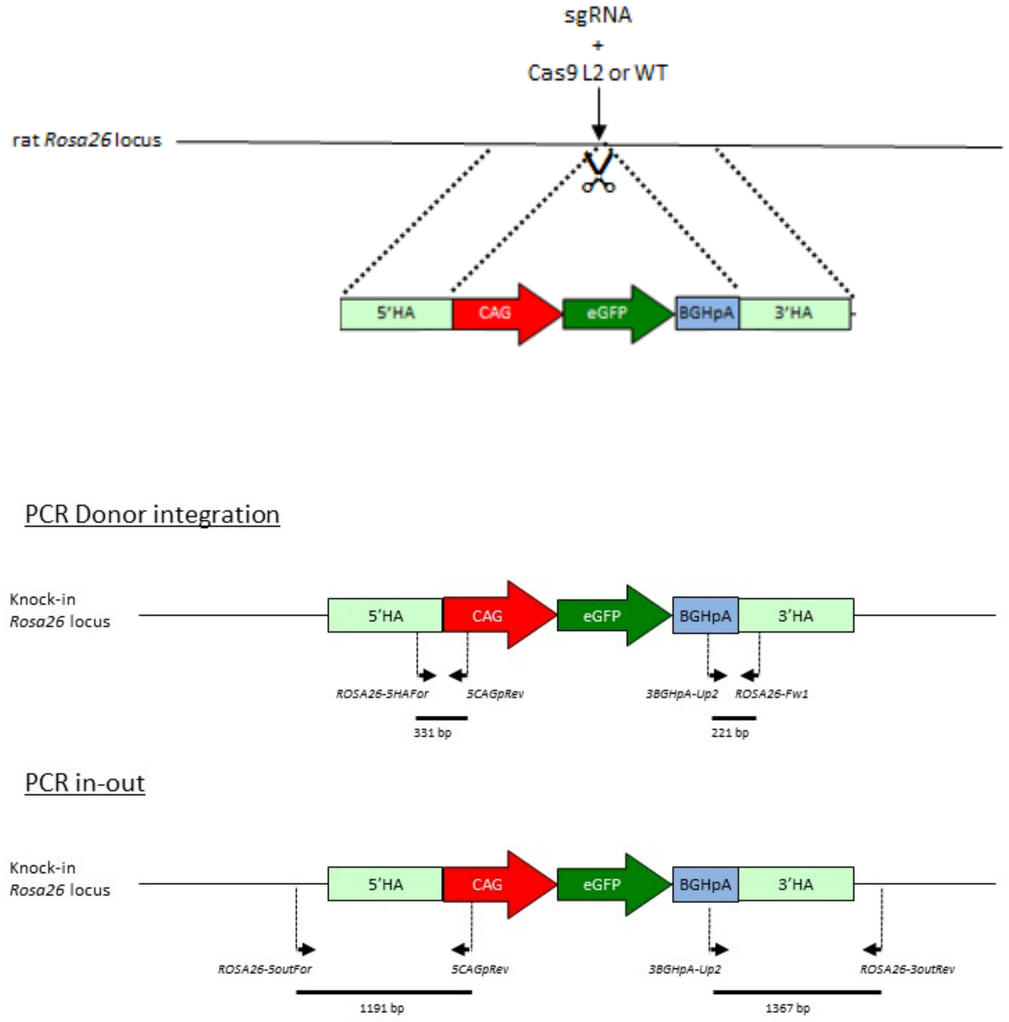

Supplementary Figure 5. PCRs performed to genotype rat embryos following microinjection into rat eggs.

The schematic (lower panel) shows the two types of PCRs used to genotype E14 rat embryos : (i) to analyze whether the insertion has occurred into the *Rosa26* locus as expected at both 5' and 3' extremities, "in-out PCR" were performed with an primer internal to the transgene sequence and a primer corresponding to genomic sequences that are beyond the homology arms of the donor sequence (ROSA26-5outFor and ROSA26-3outRev) (ii) to analyze for donor integration, random or targeted, only primers internal to the donor were used. PCRs using primers rROSAfw1 and rROSArev1 allowed to identify embryos with no donor DNA insertion but with indels at the guide RNA target sequence. A similar PCR strategy was used to analyze donor integration at the *Il22bp* locus. PCR primer sequences are given in supplementary Table 1.

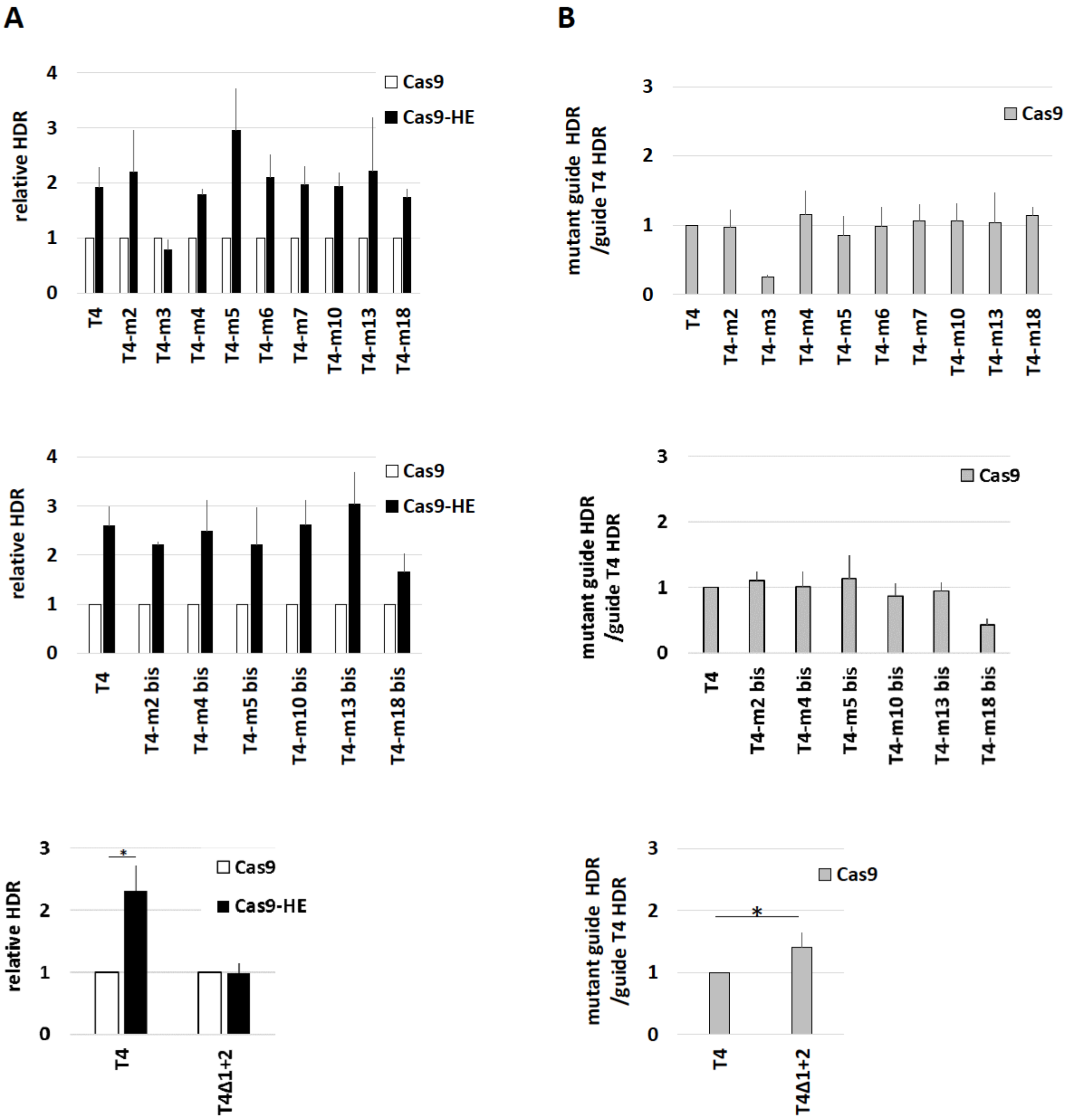

C

	guide sequence	PAM
**T4**	GACAGAAAAGCCCCATCCTT	AGG
**T4-m2**	GtCAGAAAAGCCCCATCCTT	AGG
**T4-m3**	GAaAGAAAAGCCCCATCCTT	AGG
**T4-m4**	GACcGAAAAGCCCCATCCTT	AGG
**T4-m5**	GACAaAAAAGCCCCATCCTT	AGG
**T4-m6**	GACAGcAAAGCCCCATCCTT	AGG
**T4-m7**	GACAGAtAAGCCCCATCCTT	AGG
**T4-m10**	GACAGAAAAaCCCCATCCTT	AGG
**T4-m13**	GACAGAAAAGCCtCATCCTT	AGG
**T4-m18**	GACAGAAAAGCCCCATCgTT	AGG
**T4-m2 bis**	GcCAGAAAAGCCCCATCCTT	AGG
**T4-m4 bis**	GACtGAAAAGCCCCATCCTT	AGG
**T4-m5 bis**	GACAcAAAAGCCCCATCCTT	AGG
**T4-m10 bis**	GACAGAAAAcCCCCATCCTT	AGG
**T4-m13 bis**	GACAGAAAAGCCgCATCCTT	AGG
**T4-m18 bis**	GACAGAAAAGCCCCATCaTT	AGG
**T4Δ1+2**	CAGAAAAGCCCCATCCTT	AGG

D

HDR frequencies

	exp. n1		exp. n2		exp. n3	
	Cas9	Cas9-HE	Cas9	Cas9-HE	Cas9	Cas9-HE
T4	4.4	6.7	3.4	7.5	13.3	27.2
T4-m2	3.1	9.5	3.4	6.4	15.9	26.3
T4-m3	1	0.6	0.9	0.8	3.7	3.3
T4-m4	3.8	6.8	5.2	8.7	14.2	26.8
T4-m5	2.6	9.5	2.7	8.2	15.3	32.9
T4-m6	3	6.2	3.6	9.1	16.2	27.9
T4-m7	4	9.1	3.2	6.4	17.8	28.9
T4-m10	3.8	8.4	3.4	6.4	17.7	30
T4-m13	2.5	8.3	3.9	7	18.7	28.8
T4-m18	4.6	7.5	4.3	7.3	14.9	28.3

	exp. n1		exp. n2		exp. n3	
	Cas9	Cas9-HE	Cas9	Cas9-HE	Cas9	Cas9-HE
T4	10.8	25	6.7	20.4	2.4	5.8
T4-m2 bis	10.6	22.7	7.3	16.1	3	6.8
T4-m4 bis	8.4	26.9	6.7	15	3	6.1
T4-m5 bis	9.6	27.2	6.5	15.8	3.7	5.1
T4-m10 bis	7	22.2	6.7	14.7	2.3	5.7
T4-m13 bis	10.9	29.8	7	18.1	1.9	7.2
T4-m18 bis	4.1	6.6	2.4	4.9	1.3	1.7

Indel frequencies

	exp. n1		exp. n2		exp. n3	
	Cas9	Cas9-HE	Cas9	Cas9-HE	Cas9	Cas9-HE
T4	11.3%	7.2%	3.2%	3.1%	14.1%	9.4%
T4-m2	12.5%	12.8%	6.8%	4.4%	17.8%	19.7%
T4-m3	3.9%	2.1%	3.3%	0.7%	5.3%	2.4%
T4-m4	12.2%	12.6%	8.1%	7.7%	18.6%	17.0%
T4-m5	14.5%	12.8%	6.2%	5.1%	17.6%	16.5%
T4-m6	15.6%	9.8%	6.7%	6.0%	18.6%	18.2%
T4-m7	15.4%	11.3%	5.0%	5.7%	20.2%	18.2%
T4-m10	16.3%	9.5%	4.0%	5.1%	22.7%	19.5%
T4-m13	15.1%	12.6%	5.7%	3.6%	21.6%	19.0%
T4-m18	14.8%	9.2%	5.5%	4.7%	14.0%	13.8%

	exp. n1		exp. n2		exp. n3	
	Cas9	Cas9-HE	Cas9	Cas9-HE	Cas9	Cas9-HE
T4	20.2%	14.3%	8.7%	8.5%	16.7%	11.9%
T4-m2 bis	23.0%	12.2%	10.0%	7.4%	19.5%	12.3%
T4-m4 bis	22.7%	25.2%	12.2%	8.8%	22.1%	15.6%
T4-m5 bis	25.8%	19.9%	10.0%	9.7%	21.8%	15.8%
T4-m10 bis	14.7%	12.5%	7.6%	7.6%	15.7%	11.4%
T4-m13 bis	23.0%	22.9%	11.8%	9.4%	18.7%	16.2%
T4-m18 bis	4.5%	2.6%	2.7%	1.8%	4.4%	2.5%

HDR frequencies

	exp. n1		exp. n2		exp. n3		exp. n4		exp. n5		exp. n6	
	Cas9	Cas9-HE	Cas9	Cas9-HE	Cas9	Cas9-HE	Cas9	Cas9-HE	Cas9	Cas9-HE	Cas9	Cas9-HE
T4	10.8	25	6.7	20.4	2.4	5.8	3.7	6.9	1	2.1	4.5	9.5
T4Δ1+2	14	15.4	9.3	10.9	4	4.4	3.9	3.6	1.7	1.4	5.8	4.6

Indel frequencies

	exp. n1		exp. n2		exp. n3		exp. n4		exp. n6	
	Cas9	Cas9-HE	Cas9	Cas9-HE	Cas9	Cas9-HE	Cas9	Cas9-HE	Cas9	Cas9-HE
T4	20.0%	14.0%	8.7%	8.5%	16.7%	11.9%	18.1%	13.7%	9.5%	6.0%
T4Δ1+2	21.0%	13.0%	12.8%	7.5%	20.9%	14.3%	22.8%	14.0%	8.5%	3.5%

Supplementary Figure 6 Analysis of guide RNA T4 sequence involved in HDR stimulation

Human HEK293 cells were transfected with the indicated mutants of guide RNA T4, Cas9 or Cas9-HE and a GFP transgene donor with homology arms to the AAVS1 targeted locus. HDR-mediated transgene integration was measured by FACS analysis of GFP-positive cells resulting from targeted GFP transgene integration. Indels were measured by the T7 assay. The sequence of mutants tested is indicated in section C (the name of the mutant corresponds to the position of the mutation in the guide RNA sequence).

A) Relative frequencies of HDR induced by Cas9-HE were compared to those induced by Cas9 with the indicated mutants of guide RNA T4. Results are expressed as mean of relative HDR calculated by normalizing every HDR by that induced by Cas9. The data represented is from 3 independent experiments and raw experimental results are provided in section D here below.

B) Impact of guide RNA mutations on HDR with Cas9. Results are expressed as mean of relative HDR calculated by normalizing every HDR by that induced by guide RNA T4.

C) Sequence of mutants of guide RNA T4. Mutants are named according to the position of the mutation in the guide RNA sequence. Substitutions were chosen based on systematic studies of guide RNA mutations (Doench et al., 2016) that predicted either little impact on indel frequency (T4-m2 to T4-m18 mutants) or a stronger impact (T4-m2 bis to T4-m18 bis).

D) Raw data of HDR frequency (GFP-positive cells resulting from targeted GFP transgene integration) and indel frequency (as measured by the T7 assay).

A

Target	Cas9 form	number of G418-resistant colonies			
		exp.n1	exp.n2	exp.n3	exp.n4
**ATF4**	Cas9	177	151	95	70
"	Cas9-HE	332	388	300	160
**GABP**	Cas9	1200	650	1264	2176
"	Cas9-HE	1540	600	1008	2500
**TGIF2**	Cas9	not tested	118	402	1188
"	Cas9-HE	"	367	650	2224
**RAD21**	Cas9	"	216	600	260
"	Cas9-HE	"	586	1000	840
**CREB**	Cas9	"	410	244	2200
"	Cas9-HE	"	444	480	2950

B

HDR frequencies

	exp. n1		exp. n2		exp. n3		exp. n4		exp. n5	
	Cas9	Cas9-HE	Cas9	Cas9-HE	Cas9	Cas9-HE	Cas9	Cas9-HE	Cas9	Cas9-HE
T2	0.8	1.6	0.2	0.2	4.3	8.3	3.3	5.3	0.5	1
T4	2.7	5.8	0.9	2	8.6	16.1	7.6	15.3	1.1	2.8
D1	1.2	2.6	0.3	0.4	4.2	9	7	9.2	0.9	1.4
D2	5.7	4.2	1.3	0.7	11.6	8.6	12	7.8	1.9	1.3
D3	6	4.5	1	0.6	11	9.1	9.3	11.6	2.3	1.3

Indel frequencies

	exp. n1		exp. n2		exp. n3		exp. n4		exp. n5	
	Cas9	Cas9-HE	Cas9	Cas9-HE	Cas9	Cas9-HE	Cas9	Cas9-HE	Cas9	Cas9-HE
T2	11.8%	16.1%	1.6%	2.0%	13.2%	16.6%	20.8%	18.5%	16.5%	17.8%
T4	12.4%	9.2%	2.3%	2.7%	3.1%	8.3%	18.9%	20.5%	18.7%	13.1%
D1	8.8%	10.5%	1.4%	4.9%	18.5%	14.9%	28.0%	18.7%	15.6%	15.0%
D2	11.5%	6.3%	5.8%	4.5%	18.2%	8.6%	30.8%	22.6%	16.9%	9.9%
D3	11.2%	10.5%	3.9%	5.1%	19.6%	14.4%	28.4%	31.3%	20.0%	14.7%

Supplementary Figure 7 Raw data from experiments reported in Figure 5 is given here.

A) HDR frequencies induced by Cas9-HE were compared to those induced by Cas9 at 5 different target genes in HEK293 cells using previously published guide RNAs and donor plasmids (Savic et al., 2015). Targeted integration of donor plasmid results in in frame-insertion of E2A-neoR cDNA (Savic et al., 2015). G418(neomycin)-resistant colonies were counted after Cresyl violet staining to measure HDR-mediated events and the number of G418-resistant colonies is given here for each experiment. Data is from 3 independent experiments for TGIF2, RAD21, and CREB genes and from 4 for ATF4 and GABP genes.

B) HDR and indel frequencies induced by Cas9-HE were compared to those induced by Cas9 with the indicated guide RNAs, which all target cleavage to a small 50 bp region of the AAVS1 locus, and a common p84Δ donor plasmid, harboring approximately 800 bp homology arms. P84Δ was derived from the p84 donor plasmid depicted in Fig1A by shortening the homology arms so that they would not be cleaved by any of the guide RNAs. Data represented is from 5 independent experiments. The HDR and indel frequencies (corresponding to the percentage of GFP-positive cells and indel frequencies measured with the T7 assay respectively) are given

here without the normalization that was performed to represent data in Figure 5B. Absolute levels of HDR and indel frequencies varied significantly between independent replicate experiments shown here. This variation appeared to be influenced at least in part by cotransfection efficiency. Transfection efficiency was measured in parallel by transfection of GFP expression plasmid into cells of an independent well (GFP-positive cells measured by FACS: exp.n1, 93%; n2, 75%; n3, 99%; n4, 99%, n5,86%). We noted that in the data of the table in 5B, the highest HDR frequencies obtained for each guide with Cas9 or Cas9-HE (exp. n3 and n4) corresponded to the highest transfection efficiencies (99% GFP positive cells). Higher transfection efficiencies are likely important for most efficient cotransfection of all three plasmids (which is a prequisite for HDR to take place). Other parameters may come into play such as cell cycle stage or specific optimization of the donor construct to each guide RNA cleavage site but were not investigated.

**Primers for analysis of ROSA locus targeting**	**5'-3' Sequence**
NHEJ detection<br>rROSAfw1<br>rROSArev1	 TGAACTGTGAATAGGCCCAAGTG<br>GCATTTTAAAAGAGCCCAGTACTTCA
Donor insertion<br>GFP Up<br>GFP Lo3	 CCTCGTGACCACCCTGACCT<br>TCCATGCCGAGAGTGATCCC
In/out PCR<br>rROSA26-5outFor<br>5CAGpRev<br><br>3BGHpA-Up2<br>rROSA26-3outRev	 TCCCACCCTCCCCTTCCTCT<br>GGCTATGAACTAATGACCCCGTAAT<br><br>CCAGATTTTTCCTCCTCTCCTG<br>TGGGTATCACTGGCTGTCCTAGATA

**Primers for analysis of IL22BP locus targeting**	**5'-3' Sequence**
NHEJ detection<br>Up2 64-rIL22bp<br>Lo2 64-rIL22bp	 CCATCCAGGAGACATCCTGTGGCGCA<br>CCAGGCTGGCCCTAAAGACAGA
Donor insertion<br>GFP Up<br>GFP Lo3	 CCTCGTGACCACCCTGACCT<br>TCCATGCCGAGAGTGATCCC
In/out PCR<br>rIL22BP5HAOutFor<br>GFP Lo3<br><br>GFP Up<br>rIL22BP5HAOutRev	 AAGCTCCTTCTGTCATTACTGCCT<br>TCCATGCCGAGAGTGATCCC<br><br>CCTCGTGACCACCCTGACCT<br>ACCCTTTCCAGCATGTTCTTCC

Supplementary table 1.
Primers for PCR analysis of transgene integration at the ROSA and IL22BP loci in rat.

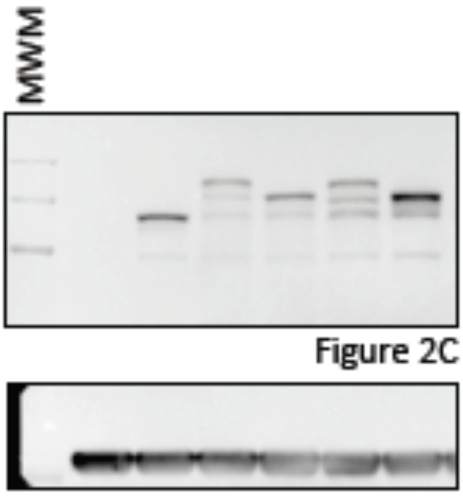

Figure 2C

Figure 2C

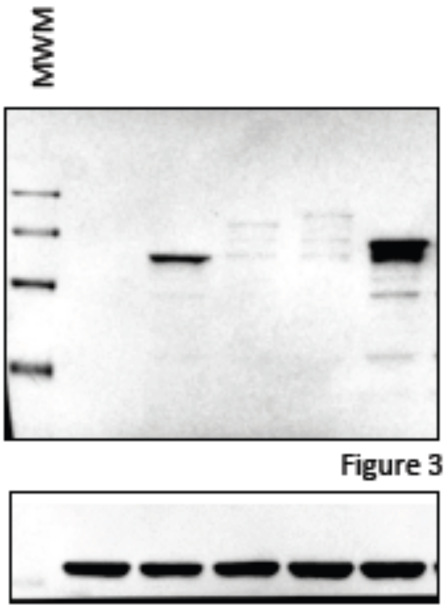

Figure 3

Figure 3

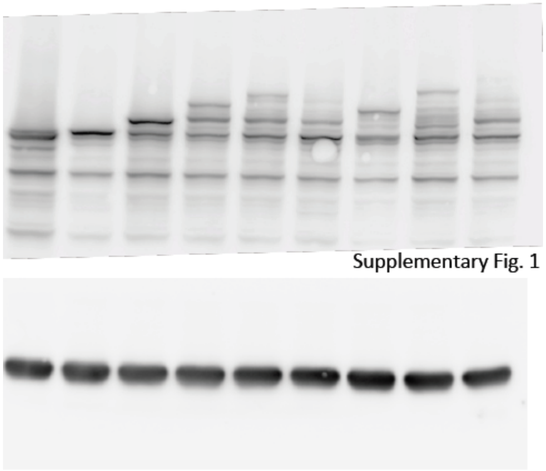

Supplementary Fig. 1

Supplementary Fig. 1

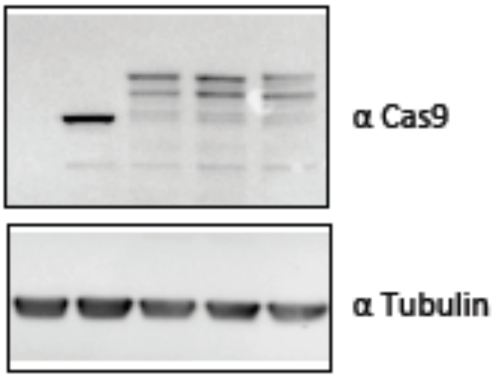

Supplementary Fig. 2B

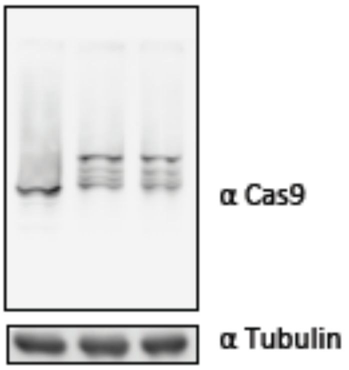

Supplementary Fig. 2C

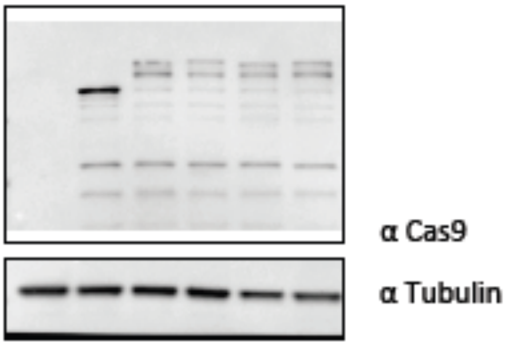

Supplementary Fig. 2D

Supplementary Figure 8. Uncropped western blots.
